# Improving stamina and mobility with preop walking in surgical patients with frailty traits –OASIS IV: randomized clinical trial study protocol

**DOI:** 10.1186/s12877-020-01799-y

**Published:** 2020-10-07

**Authors:** Laboni Hoque, Ryan Dewolf, David Meyers, Daniel K. White, Kathleen M. Mazor, Mihaela Stefan, Sybil Crawford, Karim Alavi, Jennifer Yates, Mark Maxfield, Feiran Lou, Karl Uy, Matthias Walz, Alok Kapoor

**Affiliations:** 1 University of Massachusetts Medical School, 365 Plantation St, Worcester, MA 01605 USA; 2 University of Massachusetts Memorial Health Care, Worcester, MA USA; 3 University of Delaware, Newark, Delaware USA; 4 Meyers Primary Care Institute, a joint endeavor of University of Massachusetts Medical School, Reliant Medical Group, and Fallon Health, Worcester, MA USA; 5 Baystate Medical Center, Springfield, MA USA

**Keywords:** Frailty, Surgery, Prehabilitation, Stamina

## Abstract

**Background:**

Frail older surgical patients face more than a two-fold increase in postoperative complications, including myocardial infarction, deep vein thrombosis, pulmonary embolism, pneumonia, ileus, and others. Many of these complications occur because of postoperative loss of stamina and poor mobility. Preoperative exercise may better prepare these vulnerable patients for surgery. We present the protocol for our ongoing randomized trial to assess the impact of a preoperative walking intervention with remote coaching and pedometer on outcomes of stamina (six-minute walk distance- 6MWD) and mobility (postoperative steps) in older adults with frailty traits.

**Methods:**

We will be conducting a randomized clinical trial with a total of 120 patients permitting up to a 33% rate of attrition, to reach a final sample size of 80 (with 40 patients for each study arm). We will include patients who are age 60 or higher, score 4 or greater on the Edmonton Frailty Scale assessment, and will be undergoing a surgical operation that requires a 2 or more night hospital stay to be eligible for our trial. Using block randomization stratified on baseline 6MWD, we will assign patients to wear a pedometer. At the end of three baseline days, an athletic trainer (AT) will provide a daily step count goal reflecting a 10–20% increase from baseline. Subsequently, the AT will call weekly to further titrate the goal or calls more frequently if the patient is not meeting the prescribed goal. Controls will receive general walking advice. Our main outcome is change in 6MWD on postoperative day (POD) 2/3 vs. baseline. We will also collect 6MWD approximately 4 weeks after surgery and daily in-hospital steps.

**Conclusion:**

If changes in a 6MWD and step counts are significantly higher for the intervention group, we believe this will confirm our hypothesis that the intervention leads to decreased loss of stamina and mobility. Once confirmed, we anticipate expanding to multiple centers to assess the interventional impact on clinical endpoints.

**Trial registration:**

The randomized clinical trial was registered on clinicaltrials.gov under the identifier NCT03892187 on March 27, 2019.

## Background

Frail older surgical patients face more than a two-fold increase in postoperative complications, including myocardial infarction, deep vein thrombosis, pulmonary embolism, pneumonia, ileus, and others compared with non-frail older adults [1]. Many of these complications occur because of postoperative loss of stamina and poor mobility. Preoperative exercise interventions (i.e., prehabilitation) may better prepare these vulnerable patients for surgery, but there have been few published studies focused on them. Existing interventions [2–4] fall short of meeting the needs of frail older adults because they included several clinic visits which add to the stress of these patients who have multiple other pre-surgical appointments. Most interventions included general walking advice but no goal setting with modern pedometers or remote coaching, both of which have been effective in other settings [5, 6]. In this paper, we present the protocol for our ongoing randomized trial to assess the impact of a preoperative walking intervention with remote coaching and a modern pedometer on outcomes of stamina (6MWD) and mobility (postoperative step counts) in older adults with frailty traits. The randomized clinical trial is a parallel group trial with 1:1 allocation in intervention and control groups, designed to test the superiority of a supervised prehabilitation walking program in comparison with no directed prehabilitation before a major surgery. Intervention patients will participate in a supervised preoperative walking program, while control patients will receive only general walking advice once at the time of recruitment so we may evaluate the impact of our program. We expect intervention patients to show improved postoperative mobility and greater recovery of stamina compared to their baseline status in comparison to control patients.

## Methods

### Population and setting

We will identify adults 60 years and older who are being scheduled for a major operation within 3–8 weeks, requiring a 2+ night hospital stay, and with frailty traits defined as those scoring ≥4 on the Edmonton Frailty Scale [7]. We chose this score threshold given previous research suggesting that patients scoring at this level were vulnerable to postoperative complications [8]. Originally, we intended to recruit patients undergoing colorectal surgery alone, but we found it challenging to identify sufficient patients who met eligibility criteria during the test phase-i.e. surgical plan confirmed with more than 3 weeks but less than 8 weeks. As a result, we have expanded recruitment to include patients from ENT, surgical oncology, thoracic surgery, transplant, and urology practices. We will approach eligible patients and will obtain written consent 3–8 weeks before their surgical date to ensure adequate time to complete the intervention. We will exclude patients unable to walk independently and those who have fallen in the past 3 months due to loss of balance. We will not exclude patients who are receiving concomitant physical therapy or patients participating in other, non-exercise related studies. We will be recruiting all patients from a large health care system in central Massachusetts.

### Procedures

#### Recruitment

We will screen appointment schedules and meet with eligible patients in person at least 3-8 weeks before their planned surgical date after introduction by the treating provider (usually at the final visit when the surgical plan will be established and surgery scheduling will occur). We will then administer the Edmonton Frailty Scale and then obtain written consent and HIPAA authorization for patients who may score ≥ 4.

#### Baseline interview

**Fig. 1:**
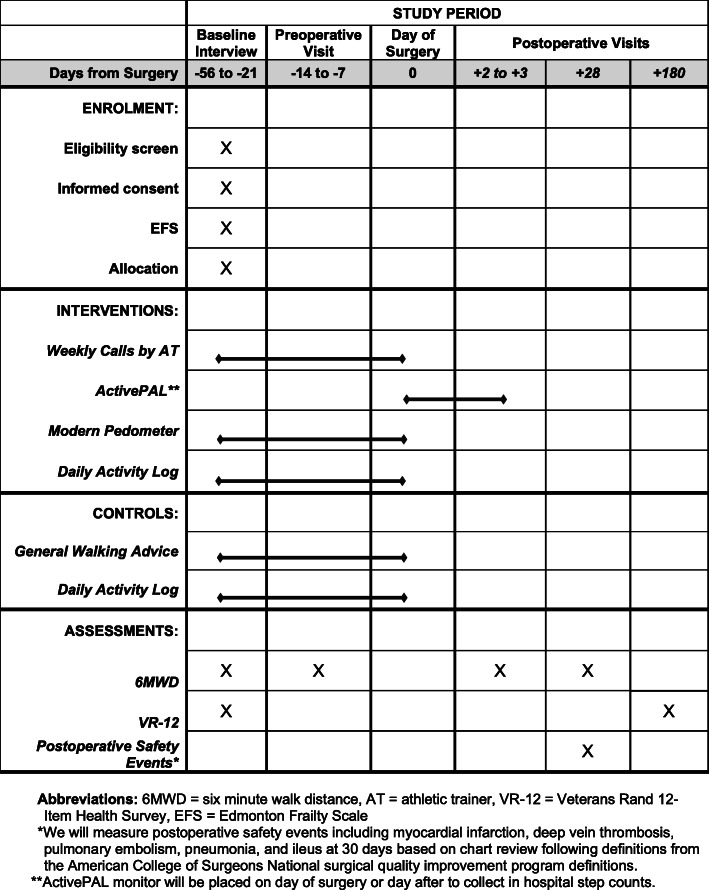
Timeline of Recruitment and Measurement Procedures

(Figure 1) We will ask patients to complete the Veterans Rand 12-Item Health Survey (VR-12) to assess their self-perceived health. The patients will then complete a 6MWD following published guidelines [9].

#### Randomization

We will randomize patients to either intervention or control using a block randomization scheme stratified on the baseline 6MWD categories informed by our test phase: 0-200 m, 201-300 m, 300-400 m, and 400 + m. REDCap software will be used to generate the randomization sequence. After completing the baseline interview, the recruiter will input the baseline 6MWD and inform the patients of the group assignment generated by REDCap.

#### Intervention protocol

We will provide each intervention patient a Garmin Vivofit4 pedometer and smart phone linked by Bluetooth to the pedometer.

During device orientation, we will teach patients how to synchronize the pedometer to the phone so that an athletic trainer (AT) can perform remote coaching and review step counts every day on the vendor’s Garmin Connect website. To develop a walking step count goal, we will ask patients to walk their usual amount for 3 days following the baseline interview.

On the fourth day after the baseline interview, the AT will call the patient and prescribe an initial step count goal based on the average of the 3 baseline days increased by 10–20%. The AT subsequently will provide weekly counseling calls to all patients, review any difficulties with the patient and, prescribe the next week’s daily step count goal. The AT will keep the same goal, if the patient has not met the previous week’s goal. The AT will also increase the daily step count goal by 10–20% if the patient meets or exceeds the goal. In addition to weekly check-in phone calls, the AT will also make interim calls whenever a patient falls below the daily step count target over two consecutive days to troubleshoot. During these calls, the AT will evaluate the patient’s comfort level with the ongoing intervention. Patients will be removed from the trial if they are injured or seem to be express unreasonable distress during calls.

#### Control patients

We will provide general walking advice but not the pedometer or remote coaching.

#### Follow-up visits

We will assess both intervention and control patients at several points following the baseline and compare to the baseline measurements. All of these measurements are will be recorded in REDCap. We will measure 6MWD at their preoperative appointments, generally 1–2 weeks prior to surgery (Fig. 1).

On the day of surgery or the following morning, we will place an activPAL3 thigh worn monitor on the patient. We will remove the activPAL3 on post-operation day (POD) 2 or 3. Finally, we will measure 6MWD a third time; this third measurement forms the basis of our primary outcome. This 6MWD will provide an acute measure of how quickly the patient is recovering and will subsequently be discharged.

At the first outpatient postoperative appointment with the surgeon, generally 1 month after surgery, we will measure 6MWD a final time.

We will also conduct a chart review after all data will be collected to measure the 30-day rate of postoperative complications. These include myocardial infarction, deep vein thrombosis, pulmonary embolism, pneumonia, and ileus, following definitions we previously adapted from the American College of Surgeons National Surgical Quality Improvement Program [10].

Six months following surgery, we will call patients and administer a repeat VR-12 assessment by phone to assess any changes in self-perceived health.

If any participants choose not to complete any assessment, they will still be enrolled in the trial unless they request to be removed. We will collect all physical data they are willing to complete and will mark their refusal for measures they choose not to complete. In order to measure adherence with the intervention, we will track the number of days a patient met his/her step count goal out of the total number of days the patient will have walked.

### Outcomes

#### Primary outcome measure

Change from baseline 6MWD to 6MWD on POD 2 or 3.

#### Secondary outcome measures

1. **Steps total on POD 1 and 2 –** These steps will be counted by modern pedometer and will be steps that will be taken on POD 1 and day 2.
2. **Total difference in 6MWD from day of baseline visit and presurgical appointment.**
3. **Total difference in 6MWD from day of baseline visit to 4-week post-op visit after surgery.**
4. **Total difference in VR-12 score from baseline visit to 6-month telephone follow up visit.** – The Veterans Rand 12-Item Health Survey (VR-12) aims to assess a patients overall health status via multiple health domains ranging from their psychological to physical health status [11]. Question 1, 3–5 and 7–9 are on a five-point likert scale, questions 2a and 2b are on a 3-point yes/no scale and questions 6a-6c are on a six-point scale. These points will be pooled to determine a total score where a higher score represents a more negative perspective of one’s health [11]. Additionally, the total score will then be used to determine a physical component score (PCS) using a previously published algorithm. The algorithm defines the US population norms for PCS as a mean of 50, a range of 0 to 100 and 10 as the standard deviation [12].

#### Data monitoring committee

The University of Massachusetts Institutional Review Board (IRB) monitors all human research conducted by University of Massachusetts Medical School investigators. A committee of faculty on the IRB has reviewed and approved this study protocol. It is independent from the sponsor and competing interests. Frequency and procedures for auditing trial conduct will be at the discretion of the IRB (i.e. periodic audits of study protocols) and is independent from the investigators and the sponsors. We will seek IRB approval for any important procedure modifications and inform trial participants per their guidelines. We will also register these modifications with Clinicaltrials.gov. The research staff will report any solicited and spontaneously reported adverse events and other unintended effects of trial interventions/conduct to the IRB and manage these events according to their guidelines.

#### Covariates

Covariates include variation in baseline walking distance as recommended in the literature [13], time between study enrollment and surgery, day of recorded follow-up of 6MWD (POD2 or POD3), laparoscopic versus open surgery, chemotherapy use prior to surgery, and characteristics related to attrition (i.e., comorbidity, self-efficacy, and pain).

#### Analysis

We will compile all data and measurements on a secure REDCap server. Once all data has been collected, it will be de-identified for analysis.

Our primary analysis will be intention to treat. We will also examine differences in effect of the intervention in patients who adhered more successfully to the intervention compared with controls. More specifically, in a sensitivity analysis, we will control for percent adherence as described above.

In terms of the analysis approach, we will use analysis of covariance to compare intervention and control groups for our outcomes and adjust for covariates We will employ model selection procedures to include those covariates that are strongly related to the outcome, particularly any that are related to the walking prescription (e.g. pain limiting the amount of walking) [14].

To handle departures from a normally distributed outcome, we will identify relevant analytic approaches based on the observed distribution. If a non-negligible proportion of patients are unable to walk at all after surgery, we will compare Tobit modeling (left censoring of zero values) [15] with alternate methods [16]. If floor or ceiling effects are plausible, we will consider the approach used in Evans et al. [17] to allow the range of possible within-person change in 6MWD to vary by baseline 6MWD; briefly, we will rank within-participant changes in 6MWD, transform these ranks using normal scores, and compare the groups regarding transformed ranks using a 2-sample t-test or Wilcoxon signed rank testing.

For clinical postoperative complications, we only anticipate being able to examine for a trend of one group having fewer events than the other.

#### Sample size determination and calculation

We will recruit 120 patients permitting up to a 33% rate of attrition (to reach a final sample size of 80) from cancellation of surgery, patient withdrawal, or difficulty collecting postoperative measurements. Applying 80% power with 5% Type I error rate for 2-sided hypothesis testing and using a standard deviation of 48 m for within-patient Δ6MWD found in Gillis et al. [2], we computed that we will need follow-up outcome data on 80 patients total (40 patients per treatment arm) to detect a mean between-group difference in Δ6MWD of 30.5 m (0.63 effect size). This value is within the clinically meaningful range found in the Gillis study (45.4 m) [2].

.

## Discussion

We are conducting a randomized clinical trial to test the impact of a remote prehabilitation program targeting elderly patients with frailty traits. We recruit these subjects approximately 3–8 weeks before their surgical dates and randomize them to intervention and control groups using a block randomization scheme stratified on baseline 6MWD. Intervention patients receive targeted walking instruction from an AT and remote monitoring via pedometer whereas control patients receive only general advice to exercise, and the physical stamina of these patients is assessed at various points of their hospital course. We have a robust analysis design in place to handle potential floor effects in postoperative stamina and mobility. We have also powered our study to detect a clinically significant mean between group difference.

Several other studies have tested interventions to improve postoperative patient stamina with prehabilitation. Gillis et al. conducted a randomized trial comparing prehabilitation vs. rehabilitation in patients undergoing colonic resection for cancer and showed a meaningful improvement in postoperative exercise capability due to prehabilitation [2]. Similarly, Li et al. showed improved postoperative functional recovery following a one-month trimodal prehabilitation program before colorectal cancer surgery [3]. However, these studies were not able to show a reduction in medical complications, discharge to nursing home, or readmission. This could be due to variety of reasons such as a small sample size but also that these studies did not focus on older adults with frailty traits as we will do. Older adults with frailty traits could benefit more substantially from prehabilitation.

Other studies have also specifically targeted frail older patients for prehabilitation. Carli et al. showed that in frail patients undergoing colorectal cancer resection, multimodal prehabilitation involving exercise, nutritional, and psychological interventions did not improve 30-day postoperative outcomes [18]. Waite et al. attempted a pilot home-based prehabilitation program directed at frail patients undergoing coronary artery bypass grafting (CABG) or valve surgery, showing the potential to improve functional ability and reduce in-hospital length of stay; results of the randomized trial are still pending [19]. These studies relied on clinic visits with physical therapists or kinesiologists, which can add to the stress and cost of surgery for patients already burdened with appointments in the preoperative period. Moreover, the preoperative time span can be especially short in patients undergoing cancer resection as were many of our study patients. We are leveraging widely available technology to improve stamina and mobility and doing so in a manner more convenient and inexpensive for patients.

There are also limitations to our proposed study plan and findings. We are recruiting across multiple surgical specialties. From our test phase results, however, we found the impact of this heterogeneity appears to be less than variation in baseline stamina (6MWD). As a result, we decided to randomize patients within strata defined by baseline stamina. We also encountered difficulty collecting 6MWD on POD2/3 given conflict with postoperative complications and fatigue with refusal to attempt walking. Even in the patients willing to attempt walking, we found many patients could only walk a short distance during testing. To address this floor effect in our analysis of randomized patients, we plan to examine both the distribution of Δ6MWD as well as the rank Δ6MWD of intervention vs. controls. Although we considered omitting Δ6MWD in hospital, we felt the variability in-patient rehabilitation and access to skilled nursing facility would confound our intervention if we looked at 1-month postop Δ6MWD only.

In summary, we have developed a feasible intervention to study the effect of a preoperative walking intervention bolstered by remote coaching and goal setting by modern pedometer. We have accounted for heterogeneity of surgery type and developed a sophisticated plan for analyzing postoperative 6MWD, accounting for potential floor effects. If the results of our randomized trial indicate beneficial effects of our intervention on stamina and mobility, we hope to perform a multicenter trial and assess the impact of the intervention on clinical endpoints and length of stay during surgical hospitalization.

## Data Availability

Not applicable.

## Abbreviations

6WMD: Six minute walk distance
AT: Athletic trainer
POD: Postoperative day
ENT: Ear, nose, and throat
